# Arc ubiquitination regulates endoplasmic reticulum-mediated Ca^2+^ release and CaMKII signaling

**DOI:** 10.3389/fncel.2023.1091324

**Published:** 2023-03-14

**Authors:** Mohammad A. Ghane, Wei Wei, Dina W. Yakout, Zachary D. Allen, Cassandra L. Miller, Bin Dong, Jenny J. Yang, Ning Fang, Angela M. Mabb

**Affiliations:** ^1^Neuroscience Institute, Georgia State University, Atlanta, GA, United States; ^2^Center for Behavioral Neuroscience, Georgia State University, Atlanta, GA, United States; ^3^Department of Chemistry, Georgia State University, Atlanta, GA, United States; ^4^Center for Diagnostics and Therapeutics, Georgia State University, Atlanta, GA, United States

**Keywords:** Arc, CaMKII, endoplasmic reticulum, calcium, ubiquitination

## Abstract

Synaptic plasticity relies on rapid, yet spatially precise signaling to alter synaptic strength. Arc is a brain enriched protein that is rapidly expressed during learning-related behaviors and is essential for regulating metabotropic glutamate receptor-mediated long-term depression (mGluR-LTD). We previously showed that disrupting the ubiquitination capacity of Arc enhances mGluR-LTD; however, the consequences of Arc ubiquitination on other mGluR-mediated signaling events is poorly characterized. Here we find that pharmacological activation of Group I mGluRs with S-3,5-dihydroxyphenylglycine (DHPG) increases Ca^2+^ release from the endoplasmic reticulum (ER). Disrupting Arc ubiquitination on key amino acid residues enhances DHPG-induced ER-mediated Ca^2+^ release. These alterations were observed in all neuronal subregions except secondary branchpoints. Deficits in Arc ubiquitination altered Arc self-assembly and enhanced its interaction with calcium/calmodulin-dependent protein kinase IIb (CaMKIIb) and constitutively active forms of CaMKII in HEK293 cells. Colocalization of Arc and CaMKII was altered in cultured hippocampal neurons, with the notable exception of secondary branchpoints. Finally, disruptions in Arc ubiquitination were found to increase Arc interaction with the integral ER protein Calnexin. These results suggest a previously unknown role for Arc ubiquitination in the fine tuning of ER-mediated Ca^2+^ signaling that may support mGluR-LTD, which in turn, may regulate CaMKII and its interactions with Arc.

## 1. Introduction

Synaptic plasticity relies on rapid, spatially precise signaling to appropriately alter synaptic strength. mGluR-LTD is a protein synthesis-dependent form of plasticity that is initiated by activation of G_q_-coupled receptor pathways and is canonically associated with α-amino-3-hydroxy-5-methyl-4-isoxazolepropionic acid (AMPA) receptor endocytosis (Abe et al., 1992; Aramori and Nakanishi, 1992; Palmer et al., 1997; Snyder et al., 2001; Wilkerson et al., 2018). While the involvement of the endoplasmic reticulum (ER) in regulating Ca^2+^ release to support cerebellar LTD is well established, the role of ER-mediated signaling events in regulating mGluR-LTD in the hippocampus is not (Wilkerson et al., 2018). It is established that neurons contain highly complex continuous ER networks, with ER morphology increasing in complexity at dendritic branchpoints and [Ca^2+^]_ER_ varying at different compartments (Terasaki et al., 1994; Spacek and Harris, 1997; Cui-Wang et al., 2012; Krijnse-Locker et al., 2017). Furthermore, the distribution of ER receptors that efflux Ca^2+^ (e.g., IP_3_Rs) vary throughout the neuron, with higher levels found at proximal regions (Sharp et al., 1993; Blaustein and Golovina, 2001). It is hypothesized that these various gradients offer an additional layer of local protein synthesis and receptor trafficking regulation.

Activity-regulated cytoskeleton-associated protein (Arc) is vital for numerous forms of synaptic plasticity, including mGluR-LTD (Lyford et al., 1995; Chowdhury et al., 2006; Plath et al., 2006; Shepherd et al., 2006; Beique et al., 2011; Korb and Finkbeiner, 2011; Jenks et al., 2017; Pastuzyn and Shepherd, 2017; Mabb and Ehlers, 2018; Newpher et al., 2018). Arc induction is spurred by learning and memory related behaviors, including spatial memory acquisition, consolidation, and retrieval (Guzowski et al., 1999; Ramirez-Amaya et al., 2005; Plath et al., 2006; Bramham et al., 2008; Nakayama et al., 2015). Importantly, Arc is rapidly degraded largely through proteasome- and lysosome-dependent processes (Rao et al., 2006; Yan et al., 2018). Proteasome-dependent Arc degradation is facilitated by ubiquitination of two tandem lysine residues (268/269) catalyzed by the E3 ubiquitin ligase RNF216 (Mabb et al., 2014). We previously generated a transgenic mouse that contained mutations in the Lysine sites 268/269 to Arginine (ArcKR), which resulted in Arc build up while retaining endogenous control of its expression. Hippocampal neurons isolated from ArcKR mice show DHPG-induced increases in AMPA receptor endocytosis that is associated with enhanced mGluR-LTD. ArcKR mice also have selective deficits in spatial reversal learning, including a bias in utilizing inefficient search strategies (Wall et al., 2018).

Calcium/calmodulin-dependent protein kinase II (CaMKII) is another hub of synaptic plasticity known to be essential for learning (Coultrap et al., 2014; Murakoshi et al., 2017; Rossetti et al., 2017; Bayer and Schulman, 2019). CaMKII activity is tightly regulated by extra- and intracellular Ca^2+^ dynamics, resulting in autophosphorylation at specific threonine residues that determine activity states (Glazewski et al., 2000; Elgersma et al., 2002; Gillespie and Hodge, 2013). Previous work has shown that CaMKII plays a role in mGluR-LTD (Mockett et al., 2011; Cook et al., 2022) and that dysregulation of CaMKII leads to LTD enhancement and alterations in spatial search strategies (Bach et al., 1995). Arc interacts with CaMKII, which is believed to recruit Arc to different subdomains (Donai et al., 2003; Okuno et al., 2012). Recent evidence indicates that CaMKII phosphorylates the GAG domain of Arc, which minimizes Arc oligomerization. Defects in CaMKII-dependent Arc phosphorylation increase Arc oligomerization and enhance mGluR-LTD magnitude (Zhang et al., 2019; Zhang and Bramham, 2020).

Given the importance of Arc mRNA and protein localization to specific neuronal subregions (Steward et al., 1998; Moga et al., 2004; Chowdhury et al., 2006; Peebles et al., 2010; Okuno et al., 2012), it remains to be seen whether the confluence of ER-Ca^2+^ gradients, CaMKII regulation of Arc localization and function, and the temporal dynamics of Arc could act as a mechanism for mGluR-LTD regulation and subsequent outputs. Furthermore, given that elevations of Arc are observed in several models of neuropsychiatric disease states along with dysregulation of LTD, determining the intracellular mechanisms that connect these phenomena is of great interest (Auerbach and Bear, 2010; Greer et al., 2010; Wu et al., 2011).

Here, we used the disrupted Arc ubiquitination mouse model (ArcKR) (Wall et al., 2018) to investigate these relationships. Using a previously characterized ER-localized Ca^2+^ sensor (G-CatchER^+^), we found that pharmacological activation of Group I mGluRs with S-3,5-dihydroxyphenylglycine (DHPG) increased ER-Ca^2+^ release in primary hippocampal neurons. Importantly, primary hippocampal neurons isolated from ArcKR mice showed an excess of ER-Ca^2+^ release upon DHPG treatment that occurred in a subregion-dependent manner when compared to wildtype (WT) littermate control neurons. Overexpression of the ArcKR mutation in HEK293 cells increased Arc self-assembly compared to overexpressed WT Arc. Co-expression of ArcKR with CamKII in HEK293 cells also led to enhanced interactions with activated versions of CaMKII compared to wildtype (WT) Arc. However, expression of CaMKII or mutating the CaMKII-dependent Arc phosphorylation site at S260 did not affect Arc ubiquitination. Furthermore, Arc-CaMKII colocalization varied throughout the neuron, which was altered in ArcKR neurons. Collectively, our findings suggest a role for Arc ubiquitination in regulating ER Ca^2+^ dynamics, Arc self-assembly, and CaMKII interactions.

## 2. Materials and methods

### 2.1. Animals

All animal care and use were carried out in accordance with the *National Institutes of Health Guidelines for the Use of Animals* using protocols approved by the Georgia State University Institutional Animal Care and Use Committee. Arc has been previously demonstrated to be ubiquitinated on K268 and K269 by the ubiquitin ligases RNF216 and UBE3A (Greer et al., 2010; Mabb et al., 2014). To mutate the ubiquitination sites of Arc, the conventional knock-in point mutation strategy was used where two point mutations were created within *Exon 1* of the *Arc* gene resulting in the substitution of Lysine for Arginine at amino acid positions 268 and 269. The generation of this mouse did not disrupt Arc induction from its endogenous promoter but did selectively decrease the turnover of synthesized Arc protein due to its inability to be efficiently ubiquitinated on K268 and K269 (Wall et al., 2018). Homozygous *Arc^WT/WT^* (WT) and *Arc^KR/KR^* (KR) littermates were used for all experiments and were generated by crossing heterozygous *Arc^WT/KR^* x *Arc^WT/KR^* mice using the trio breeding method.

### 2.2. Cell culture

Primary hippocampal neuron cultures were generated from postnatal day 0-1 WT and ArcKR littermates as previously described (Wall et al., 2018). Neurons were grown on poly-D-lysine (0.02 mg/ml)-coated 6-well plates (9.6 cm^2^) at a density of 300,000 cells/well for Western blotting applications; on PDL-coated (0.1 mg/ml) coverslips (12 mm diameter) in 24-well plates (3.5 cm^2^) at a density of 75,000 cells/well for immunocytochemistry (ICC) applications; and on PDL-coated (0.1 mg/ml) coverslips (24 mm × 60 mm) in 60 mm culture dishes at a density of 75,000 cells/dish for live imaging experiments. Neurons were maintained in neuronal feeding media Neurobasal media, (Gibco #12348017) containing 1% GlutaMAX (Gibco #35050061), 2% B-27 (Gibco #17504044), 4.8 μg/ml 5-Fluoro-2’-deoxyuridine (Sigma #F0503), and 0.2 μg/ml Gentamicin (Gibco #15710064) and fed by replacing half of the feeding media with fresh, pre-warmed neuronal feeding media every 3–4 days until used for experiments as described below.

HEK293 cells (ATCC) were seeded and maintained using standard procedures in Dulbecco’s modified Eagle’s medium (DMEM; Corning #10-017-CV) containing 10% FBS and 1% penicillin-streptomycin (Gibco #15240062).

### 2.3. Highly inclined laminated optical sheet (HILO) ER calcium imaging

Cultured primary hippocampal neurons were transfected with G-CatchER^+^ using Lipofectamine 2000 reagent (ThermoFisher Scientific #11668019) with a modified version of the manufacturer’s protocol as previously described (Reddish et al., 2021) at days *in vitro* (DIV) 11–12. Neurons were imaged at DIV 12–14.

To elicit and measure the magnitude of mGluR-mediated ER Ca^2+^ release, we used the type 1 mGluR agonist DHPG. G-CatchER^+^-transfected neurons were transferred to pre-warmed (37°C) artificial cerebrospinal fluid (ACSF; 124 mM NaCl, 3 mM KCl, 2 mM CaCl2, 2 mM MgCl2, 10 mM HEPES, 10 mM D-Glucose, pH 7.4). A G-CatchER^+^-positive neuron was identified and a signal was acquired at baseline. DHPG solution [100 μM (S)-3,5-DHPG (DHPG) (Tocris) in ACSF] was pre-warmed to 37°C and then washed in manually using a syringe that was connected via tubing to the imaging chamber. After imaging, the DHPG was washed out with pre-warmed ACSF.

For HILO microscopy, a fiber-coupled 488 nm laser (Oxxius) was first collimated and then focused at the back focal plane of a 100× oil TIRF objective (N.A. 1.49, Nikon) using an achromatic lens with a focal length of 200 mm (Thorlabs) as previously described (Deng et al., 2021; Reddish et al., 2021). The position of the focused laser at the back focal plane of the objective was controlled by a 2D translational stage (Thorlabs). By laterally shifting the laser beam, the incident angle of laser beam at the interface of the coverslip and cell can be easily controlled. To achieve HILO imaging, the incident angle of the laser beam at the interface was adjusted to the sub-critical angle of total internal reflection where a thin optical light sheet was refracted into cells and excited the fluorophores within a few tens of micrometers from the coverslip surface. Laser power was kept consistent at 0.7 mW to minimize photobleaching. Images were acquired with a highly sensitive electron multiplying charge-coupled device (EMCCD) camera (Andor Ixon Ultra 888) at a rate of 2 frames/s.

Live cell images were analyzed as previously described (Deng et al., 2021; Reddish et al., 2021) in ImageJ (NIH) by manually drawing a region of interest around clearly visible sub-regions of neurons [soma, primary branchpoints (PBs), primary dendrites (PDs), secondary branchpoints (SBs), and secondary dendrites (SDs)] and fluorescence traces extracted. Raw pixel values were normalized to the average baseline fluorescence to generate a profile plot. Rigid motion correction was applied in cases where the neuronal subregion drifted partially or entirely out of the drawn regions of interest using the Rigid Registration ImageJ plugin. Videos were carefully inspected to verify cell health and data discarded in cases of clear cell morphological changes that indicated cell death (severe punctuation, shriveling, or massive changes in fluorescence that were not associated with drug treatment).

Basal [Ca^2+^]_ER_ was calculated for WT and ArcKR neurons using a previously described method using pharmacological saturation (de Juan-Sanz et al., 2017; Reddish et al., 2021). Neurons were prepared and a signal was acquired as described above. Neurons were then treated with ACSF containing 50 μM ionomycin (ThermoFisher Scientific) in ACSF modified with 10 mM Ca^2+^. This saturates the G-CatchER^+^ signal, and using the known characteristics of the sensor, the following equation can be used to calculate the baseline ER Ca^2+^ concentration:


$${\left[C\,a^{2+}\right]}_{E\,R}=K_{d}\,{\left(\frac{\frac{F_{r}}{F_{m\,a\,x}}-\frac{1}{R_{f}}}{1-\frac{F_{r}}{F_{m\,a\,x}}}\right)}^{\frac{1}{n}}$$

where K_d_ is the affinity coefficient of G-CatchER^+^, F_r_ is the average measured baseline fluorescence, R_f_ is the dynamic range of G-CatchER^+^, and n is the Hill coefficient.

### 2.4. Immunocytochemistry

Cultured primary hippocampal neurons on coverslips were fixed at DIV 12–14 with 4% paraformaldehyde and 4% sucrose in phosphate-buffered saline (PBS) at 37°C, washed with PBS, permeabilized with 0.1% Triton X-100 in PBS for 15 min at room temperature, and blocked with 10% normal goat serum (Gibco #16210072) for 1 hr at 37°C. Cells were then incubated with primary antibodies [rabbit anti-Arc (Synaptic Systems #156003; 1:500); rabbit anti-CaMKIIα/β (Abcam #ab52476; 1:250)] overnight at 4°C in 3% normal goat serum in PBS, washed with PBS, and incubated with secondary antibodies [donkey anti-mouse AlexaFluor 568 (Invitrogen #A10037; 1:1000); goat anti-rabbit AlexaFluor 488 (Life Tech #A11008; 1:1000)] for 1 h at room temperature. After a final wash with PBS, coverslips were mounted onto slides with Fluorogel (GeneTex #GTX28214) or Vectashield Vibrance (Vector Laboratories #H-1800-10) hardening mounting media with DAPI.

Neurons were imaged on a Zeiss LSM 700 confocal microscope using a 63× oil immersion objective lens (NA 1.4, Zeiss #420782-9900-000). Raw z-stack images (step size 0.38 μm) were analyzed using ImageJ (NIH) by manually drawing regions of interest around clearly visible sub-regions of neurons (soma, PBs, PDs, SBs, and SDs) and the integrated density (the sum of pixel intensities in the ROI)/Area of the ROI in pixels) was measured. All parameters related to acquisition were kept constant for all images within a given dataset. For colocalization analysis, the Coloc 2 plugin (bisection threshold regression) was used to calculate above-threshold pixel-pixel Pearson’s correlation coefficients between channels.

### 2.5. Western blotting

Cultured primary hippocampal neurons were harvested by briefly washing the plates in Dulbecco’s phosphate buffered saline (Gibco #14190144) and removed from the plate using a cell scraper (Corning #3010) and pelleted by centrifugation. Cell pellets were lysed in radioimmunoprecipitation assay (RIPA) buffer (150 mM NaCl, 50 mM Tris-HCl, 1% v/v NP-40, 0.5% sodium deoxycholate, 0.1% SDS) for 30 min on ice and then centrifuged at 13,000 rpm for 20 min at 4^°^C to remove insoluble debris. Protein concentration was determined using the Pierce assay 660 assay (ThermoFisher). For primary hippocampal neuron cultures, the entire cell lysate was used without assaying protein content due to low protein content and small volume. Following separation by SDS-PAGE, proteins were transferred to a nitrocellulose membrane (0.45 μm pore size, Bio-Rad). Membranes were blocked with Intercept TBS blocking buffer (LI-COR) overnight at 4°C and then incubated with primary antibodies (Arc, Synaptic Systems #156003, 1:1000; CaMKII, Abcam #ab52476, 1:1000; CaMKII p-Thr286 p-Thr287 Novus Biologicals #NB110-96896, 1:1,000; GAPDH, GeneTex #GTX627408, 1:1,250) in a solution containing 1:1 blocking buffer:1% Tween-20 in tris-buffered saline (TBST) overnight at 4°C. After washing with TBST, membranes were incubated with secondary antibodies (IRDye Goat anti-Mouse 680RD, LI-COR #926-68070, 1:15,000, IRDye Goat anti-Rabbit 800CW, LI-COR #926-32211, 1:15,000) in 1:1 blocking buffer:TBST for 1 h at room temperature. Membranes were imaged on a LI-COR Odyssey CLx scanner (low scan quality, 163 μm scan resolution, auto channel intensities) and resulting images were analyzed using the gel analysis tool in ImageJ (NIH). For images where high background was present, background subtraction was performed on the whole channel using the Subtract Background tool in ImageJ with a rolling ball radius of 20–50 pixels.

### 2.6. Co-immunoprecipitation

HEK293 cells were seeded on 6-well plates and transfected with pRK5-myc-Arc, pRK5-myc-ArcKR, pRK5-myc-ArcKA, pEGFP-Arc, pEGFP-ArcKR, pEGFP-ArcKA, pCAG-mEGFP-CaMKIIα, and/or pCAG-mEGFP-CaMKIIβ, pCAG-mEGFP-CaMKIIα T286A, pCAG-mEGFP-CaMKIIα T286D, pCAG-mEGFP-CaMKIIβ T287A, pCAG-mEGFP-CaMKIIβ T287D, and/or mVenus-Calnexin (Addgene #56324) [CaMKII plasmids were a kind gift from Dr. Ulli Bayer at the University of Colorado Medicine (Bayer et al., 2006)] using Lipofectamine 2000 or Lipofectamine 3000 reagent (ThermoFisher Scientific) following the manufacturer procedure. After allowing constructs to express overnight, cells were harvested and lysed in co-immunoprecipitation (Co-IP) buffer (20 mM Tris-HCl, 3 mM EDTA, 3 mM EGTA, 150 mM NaCl, 1% Triton X-100, 1 mM DTT, pH 7.4) containing protease and phosphatase inhibitors [0.1 mM phenylmethylsulfonyl fluoride, 1 μM leupeptin, 0.15 μM aprotinin, and 1:2,000 Halt phosphatase inhibitor cocktail (ThermoFisher Scientific #78420)] for 30 min on ice. After centrifugation (14,000 rpm, 25 min, 4°C), lysate protein concentration was determined using a Pierce 660 assay (ThermoFisher). Ten microgram of protein was used for subsequent steps, with 1 μg of protein (10%) reserved for input control. Lysates were incubated with 0.5 μg/ml monoclonal mouse anti-myc antibody (Santa Cruz Biotech #sc-40) for 1 h at 4°C. 25 μl of pre-equilibrated GammaBind Sepharose beads (Cytiva # 17088601) were then added and samples tumbled overnight at 4°C. Control samples were incubated with pre-equilibrated GammaBind Sepharose beads. Samples were washed with Co-IP buffer and proteins were eluted from beads using SDS sample buffer (Li-Cor #928-40004) with heating at 45°C for 5 min. Subsequently, beads were pelleted by centrifugation (15,000 rpm for 2 min) and the supernatant containing protein was separated using SDS-PAGE, transferred to a nitrocellulose membrane, and immunoblotting proceeded as described above using anti-GFP (Fisher #NB600308, 1:1000) and polyclonal goat anti-myc (Bethyl/ThermoFisher #A190-104A, 1:1,000) primary antibodies. For quantification, GFP-tagged co-immunoprecipitated protein band intensities were normalized to the immunoprecipitated myc-tagged protein, which served as an important internal control for IP efficiency.

### 2.7. Generation of Arc mutations and Arc ubiquitination assay

QuickChange*™* Site-directed mutagenesis (SDM) was used to generate the CaMKII Arc phosphorylation mutants pRK5-myc-Arc S260A and pRK5-myc-Arc S260D. Primers used were as follows: S260A For.: 5′-AGTGGTGGGAGTTCAAACAGG GCGCGGTGAAGAACTGGGTGGAGTTC-3′ and S260A Rev.: 5′-GAACTCCACCCAGTTCTTCACCGCGCCCTGTTTGAACTCC CACCACT-3′; S260D For.: 5′-TGGTGGGAGTTCAAACAGG GCGACGTGAAGAACTGGGTGGAGTTC-3′ and S260D Rev.: 5′-GAACTCCACCCAGTTCTTCACGTCGCCCTGTTTGAACTCC CACCA-3′. Transfected cells were treated with 10 μM MG-132 (sigma #474790) for 4 h and then lysed in RIPA buffer with 1 mM DTT, protease and phosphatase inhibitors (0.1 mM PMSF, 1 μM leupeptin, 0.15 μM aprotinin, and 1:2,000 Halt phosphatase inhibitor cocktail; ThermoFisher #78420). Lysates were centrifuged at 13,000 rpm for 20 min at 4^°^C to remove insoluble debris. Protein concentration was determined using the Pierce assay 660 assay (ThermoFisher). One milligram of protein was used for each condition and brought up to a total volume of 1 mL in RIPA buffer. Beads (Protein A/G PLUS-Agarose; Santa Cruz #sc-2003) were pre-equilibrated in RIPA buffer with inhibitors. Protein samples were incubated with 2.5 μg/sample of the primary antibody [Goat anti-myc (Bethyl #A190-104A)] and left to tumble for 1 h at 4^°^C, then equal volume of beads suspension was added per sample and left to tumble overnight. Samples were centrifuged for 45 s at 13,000 rpm to pellet the beads. The supernatant was discarded, and beads were washed three times for 5 min with RIPA buffer before adding 2× SDS sample buffer (4% SDS, 20% glycerol, 0.2% bromophenol blue, 3% DTT, 0.1 M Tris-HCl, 1:1,000 β-mercaptoethanol, pH 6.8) and heating to 45^°^C for 5 min. Proteins were then separated using SDS-PAGE as described above and subsequent immunoblots were probed with a mouse anti-Ubiquitin (Sigma # U5379, 1:500) followed by polyclonal goat anti-myc (Bethyl/ThermoFisher #A190-104A, 1:1000) primary antibody.

### 2.8. Arc protein purification and test for multimerization

Purification of His-tagged ArcWT and ArcKR was carried out by a third-party company (GenScript Biotech, NJ, USA) using the HD transient expression of recombinant protein—gold system. Briefly, the sequences for ArcWT and ArcKR were synthesized and cloned into the pcDNA3.4 target vector using the EcoRI and HindIII restriction sites. Plasmids were then transfected into the HD 293F cell line for expression. Proteins were purified using HisTrap FF Crude (Cytiva) purification columns and eluted in PBS, pH 7.2. Purity was too low to be detected by SDS-PAGE under non-reducing conditions. After receipt, protein was further concentrated using Amicon Ultra-0.5 ml centrifugal filters (Millipore Sigma) prior to use. Proteins were brought up in the same concentration and used for subsequent SDS-PAGE analysis under reducing [SDS sample buffer containing 5% 2-mercaptoethanol (2-ME)] and nonreducing [SDS sample buffer without (2-ME)] conditions after heating for 5 min at 95°C.

### 2.9. Statistical analysis

Data are presented as mean ± SEM unless otherwise indicated. Statistical tests were performed using GraphPad Prism software. Statistical significance was defined as *p* < 0.05. For testing one independent variable when experimental groups had equal variance as determined using and F test, a *t*-test was used; otherwise, a *t*-test was used with Welch’s correction. For comparing two independent variables, a two-way ANOVA was used. To determine differences between groups following a two-way ANOVA, either Sidak’s test for multiple comparisons or Tukey’s test was used depending on the relevant comparisons. The precise statistical test used for each experiment, including sample sizes, factors, and *post-hoc* comparisons, can be found in the Results section describing the experiment.

## 3. Results

### 3.1. Excess ER calcium release in ArcKR primary hippocampal neurons

In our previous studies, CA1 field recordings from acute hippocampal slices showed that ArcKR neurons displayed enhanced mGluR-LTD in response to application of DHPG, including a reduced threshold for reaching LTD and a higher magnitude of LTD (Wall et al., 2018). Because mGluR-LTD is a G_q_ dependent process that may regulate the release of intracellular Ca^2+^ stores from the ER, we first explored the possibility that DHPG elicits altered ER Ca^2+^ release kinetics in ArcKR neurons.

**FIGURE 1:**
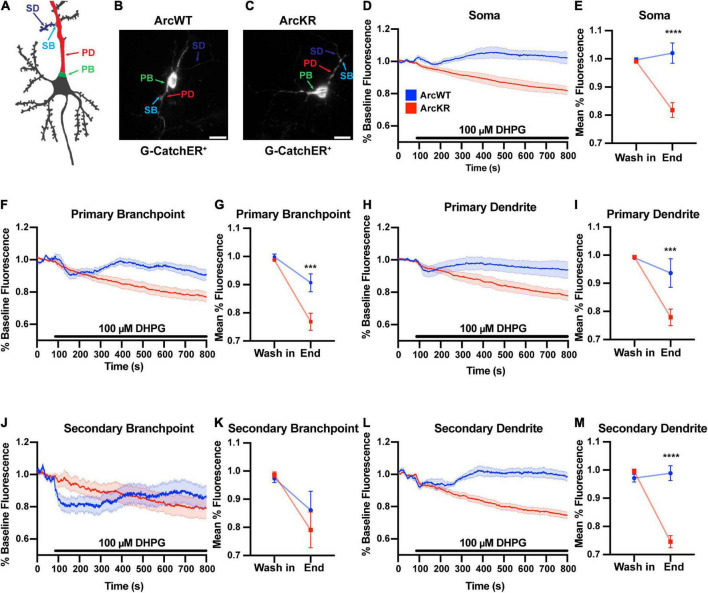
DHPG elicits excess ER Ca^2+^ release in ArcWT with disrupted dynamics in ArcKR hippocampal neurons. **(A)** Schematic of regions of interest referred to in this figure. **(B)** Representative image from a G-CatchER^+^-transfected ArcWT neuron. **(C)** Representative image from a G-CatchER^+^-transfected ArcKR neuron. **(D,F,H,J,L)** Normalized G-CatchER^+^ signal for WT (blue, *N* = 6 neurons) and ArcKR (red, *N* = 12 neurons) primary hippocampal neurons in the region of the neuron indicated with application 100 μM DHPG (black bar). Mean percent change from baseline (0–75 s) ± SEM. **(E,G,I,K,M)** Mean G-CatchER^+^ fluorescence decrease from mean baseline fluorescence (0–75 s) at wash-in of DHPG and at 800 s ArcWT (blue) and ArcKR (red) primary hippocampal neurons. Mean ± SEM. Two-way ANOVA, Soma: Genotype: *F*(1, 32) = 20.74, *p* < 0.0001, Time: *F*(1, 32) = 10.63, *p* = 0.0026, Interaction: *F*(1, 32) = 18.49, *p* = 0.0001; Sidak’s *post-hoc* test, WT vs. KR at End *p* < 0.0001; PB: Genotype: *F*(1, 32) = 9.120, *p* = 0.0049, Time: *F*(1, 32) = 39.59, *p* < 0.0001, Interaction: *F*(1, 32) = 6.651, *p* = 0.0147; Sidak’s *post-hoc* test, WT vs. KR at End *p* = 0.0008; PD: Genotype: *F*(1, 32) = 7.966, *p* = 0.0081, Time: *F*(1, 32) = 24.04, *p* < 0.0001, Interaction: *F*(1, 32) = 7.966, *p* = 0.0063; Sidak’s *post-hoc* test, WT vs. KR at End *p* = 0.0006; SB: Genotype: *F*(1, 32) = 0.3066, *p* = 0.5836, Time: *F*(1, 32) = 8.865, *p* = 0.0055, Interaction: *F*(1, 32) = 0.6407, *p* = 0.4294; SD: Genotype: *F*(1, 32) = 32.51, *p* < 0.0001, Time: *F*(1, 32) = 36.77, *p* < 0.0001, Interaction: *F*(1, 32) = 48.36, *p* < 0.0001; Sidak’s *post-hoc* test, WT vs KR at End *p* < 0.0001. Soma *n* = 6 WT, 12 KR; primary branchpoint *n* = 13 WT, 25 KR; primary dendrite *n* = 6 WT, 12 KR; secondary branchpoint *n* = 16 WT, 14 KR; secondary dendrites *n* = 14 WT, 24 KR from 4 individual culture preparations. ***p < 0.001, ****p < 0.0001, ^ns^*p* > 0.05.

ArcWT (WT) and ArcKR (KR) primary hippocampal neuron cultures were transfected with the ER-localized genetically-encoded calcium indicator G-CatchER^+^ (Reddish et al., 2021) and imaged on a highly inclined laminated optical sheet (HILO) microscope during the addition of 100 μM S-3,5-DHPG. G-CatchER^+^ is specifically targeted and expressed only in the ER lumen; thus, a decrease in G-CatchER^+^ fluorescence reflects Ca^2+^ being released from the ER (Reddish et al., 2021). As the subcellular distribution of Ca^2+^ signaling-related proteins, ER complexity, intracellular [Ca^2+^], and ER Ca^2+^ release can vary throughout the neuron (Sharp et al., 1993; Terasaki et al., 1994; Spacek and Harris, 1997; Blaustein and Golovina, 2001; Cui-Wang et al., 2012; Krijnse-Locker et al., 2017), we examined various neuronal subregions separately: soma, PBs, PDs, SBs, and SDs (Deng et al., 2021; Reddish et al., 2021; Figures 1A–C). While treatment of WT and KR neurons with DHPG caused a release of Ca^2+^ from the ER, G-CatchER^+^ fluorescence traces showed a striking depletion of ER Ca^2+^ stores in KR neurons after DHPG application (Figure 1). Moreover, ER Ca^2+^ release as quantified by percent decrease from baseline average was significantly enhanced in KR neurons at the soma (Figures 1D, E), PB (Figures 1F, G), PD (Figures 1H, I), and SD (Figures 1L, M). However, KR SB showed no difference in release compared to WT neurons (Figures 1J, K), which may be related to our previous findings that Ca^2+^ release at secondary branchpoints is reduced (Deng et al., 2021; Reddish et al., 2021).

**FIGURE 2:**
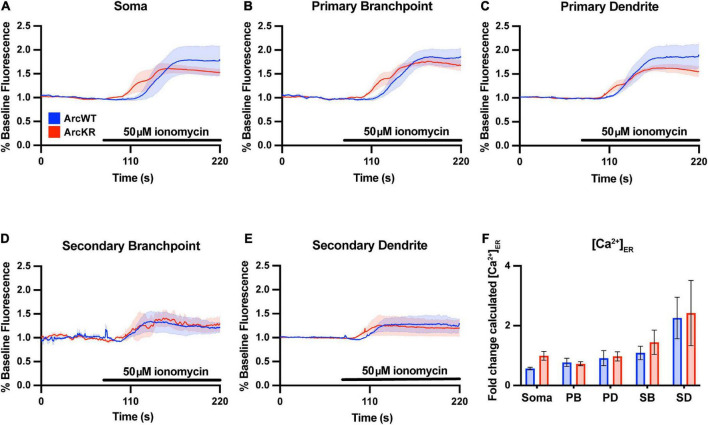
Baseline ER [Ca^2+^] is not different in ArcKR primary hippocampal neurons. **(A–E)** Mean G-CatchER^+^ fluorescence change from baseline (0–75 s) at the indicated regions during application of 50 μM ionomycin + 10 mM Ca^2+^ to ArcWT (blue, *N* = 3) and ArcKR (red, *N* = 4) primary hippocampal neurons. Mean ± SEM. Soma *n* = 3 WT, 4 KR; primary branchpoint *n* = 8 WT, 8 KR; primary dendrite *n* = 8 WT, 8 KR; secondary branchpoint *n* = 4 WT, 3 KR; secondary dendrites *n* = 3 WT, 2 KR from 2 independently prepared neuron cultures. **(F)** Calculated [Ca^2+^]_ER_ for ArcWT and ArcKR neurons. Mean ± SEM. Two-way ANOVA, Genotype: *F*(1, 41) = 1.133, *p* = 0.2935, Region: *F*(4, 41) = 7.998, *p* < 0.0001, Interaction: *F*(4, 41) = 0.2939, *p* = 0.8803.

To evaluate if the enhanced Ca^2+^ release in ArcKR neurons was simply due to baseline increases in ER Ca^2+^, we measured baseline [Ca^2+^]_ER_ during the application of the ionophore ionomycin (50 μM) in a buffer containing excess Ca^2+^ (10 mM) to saturate the G-CatchER^+^ signal and [Ca^2+^] was calculated as previously described (de Juan-Sanz et al., 2017; Figure 2). However, we found no genotype differences in basal ER Ca^2+^ in all neuronal regions examined (Figure 2F), indicating that the phenotypes observed in KR neurons were not influenced by baseline [Ca^2+^]_ER_ prior to DHPG application.

### 3.2. Altered ArcKR interaction with CaMKII depends on CaMKII isoform and phosphorylation status

**FIGURE 3:**
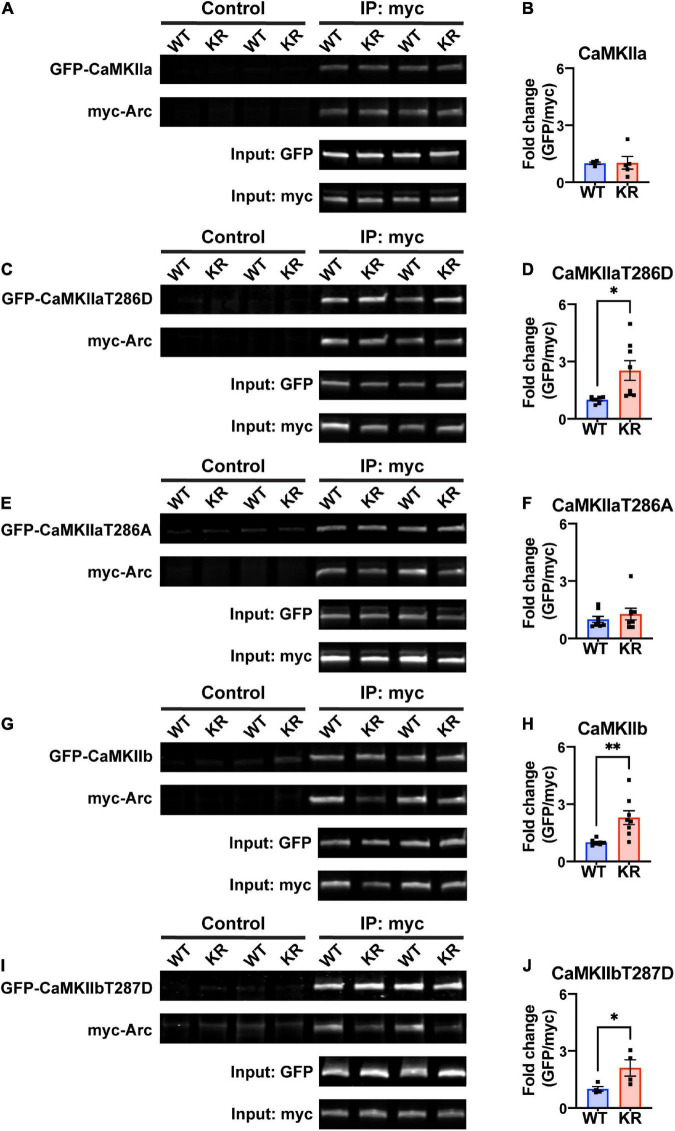
ArcKR protein differentially co-immunoprecipitates with CaMKII in an isoform- and phosphorylation status-dependent manner. **(A,C,E,G,I)** Representative Western blots after pulldown of myc-Arc and immunoblotted for the indicated tags. **(B)** Quantification of GFP-CaMKIIa after IP myc-ArcWT (blue) or myc-ArcKR (red). Unpaired *t*-test with Welch’s correction, *t* = 0.0607, df = 4.289, *p* = 0.9543. **(D)** Quantification of GFP-CaMKIIaT286D after IP of myc-ArcWT or myc-ArcKR. Unpaired *t*-test with Welch’s correction for unequal variances, *t* = 2.957, df = 7.210, *p = 0.0205. **(F)** Quantification of GFP-CaMKIIaT286A after IP of myc-ArcWT or myc-ArcKR. Unpaired *t*-test with Welch’s correction for unequal variances, *t* = 0.8050, df = 14, *p* = 0.4343. **(H)** Quantification of GFP-CaMKIIb after IP of myc-ArcWT or myc-ArcKR. Unpaired *t*-test with Welch’s correction for unequal variances, *t* = 3.564, df = 7.273, ^**^p = 0.0086. **(J)** Quantification of GFP-CaMKIIbT287D after IP of myc-ArcWT or myc-ArcKR. Unpaired *t*-test, *t* = 2.454, df = 6, *p = 0.0495. Data are represented as mean fold change relative to WT ± SEM. Each individual data point represents a single biological replicate.

Previous work has shown that Arc interacts directly with CaMKII, and that this interaction depends on the isoform and phosphorylation status of CaMKII (Okuno et al., 2012; Zhang et al., 2015, 2019). We transfected HEK293 cells with either myc-ArcWT or myc-ArcKR along with GFP-CaMKIIa or GFP-CaMKIIb. We also used CaMKII plasmids with point mutations at Thr286 (for CaMKIIa) or Thr287 (CaMKIIb) that mimic the active, autophosphorylated state (T286D/T287D) or mimic the inactive unphosphorylated state (T286A/T287A) (Bayer et al., 2006; O’Leary et al., 2006; Coultrap et al., 2012). After immunoprecipitation (IP) of Arc, protein complexes were eluted and immunoblotting proceeded for GFP- and myc-tagged proteins (Figures 3A, C, E, G, I). The interactions were quantified as described in Methods section 2.6.

While CaMKIIa-GFP interaction with ArcKR was not statistically different from WT (Figure 3B), ArcKR interaction was significantly higher with CaMKIIaT286D (Figure 3D; unpaired *t*-test with Welch’s correction, *t* = 2.957, df = 7.210, *p* = 0.0205). No difference was detected with CaMKIIaT286A (Figure 3F). Interestingly, ArcKR interaction with CamKIIb was significantly higher than WT for both WT CaMKIIb as well as CaMKIIbT287D (Figures 3H, J). These results indicate that the non-ubiquitinated form of Arc (ArcKR) interacts more highly with CaMKIIb and autophosphorylated forms of CaMKIIa and CaMKIIb.

### 3.3. ArcKR protein exhibits altered self-assembly

**FIGURE 4:**
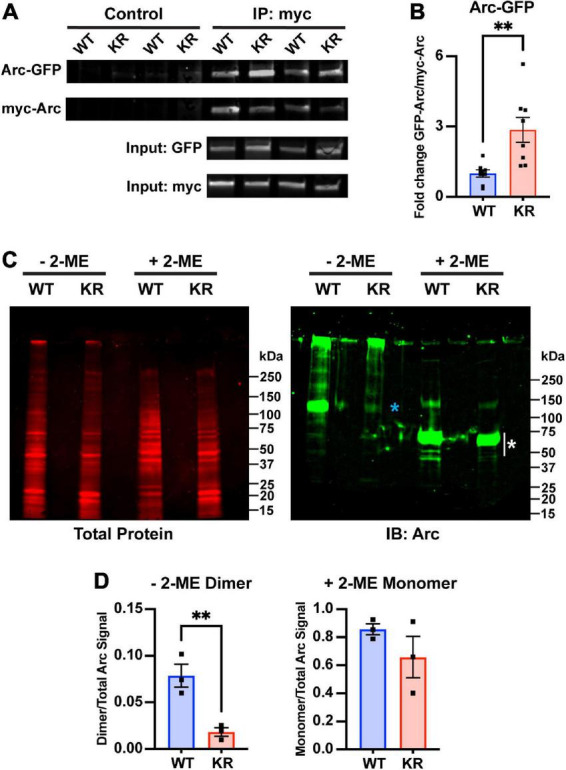
Decreased dimer formation in ArcKR. **(A)** Representative Western blots after pulldown of myc-ArcWT or myc-ArcKR with an anti-myc antibody and immunoblotted with anti-GFP to detect GFP-ArcWT or GFP-ArcKR. **(B)** Quantification of GFP-ArcWT or GFP-ArcKR after pulldown with myc-Arc or myc-ArcKR. *t*-test with Welch’s correction for unequal variances, *t* = 3.336, df = 8.199, ^**^p = 0.0099. Mean fold change relative to WT ± SEM. **(C)** His-ArcWT and His-ArcKR under non-reducing (–2-ME) and reducing conditions (+2-ME). (Left) Total protein stain for Arc. (Right) Immunoblot using an anti-Arc polyclonal antibody (blue * represents higher molecular weight portion of lane analyzed, white * represents lower molecular weight portion of lane analyzed). **(D)** Proportion of Arc signal in high molecular weight range in the non-reduced condition (left) and low molecular weight range in the reduced condition (right) normalized by the total Arc signal each lane. Unpaired t-test, non-reduced: *t* = 4.610, df = 4, *p* = 0.0100; reduced: *t* = 1.302, df = 4, *p* = 0.2629.

Arc oligomerization has been described to be negatively regulated by Ser-260 phosphorylation mediated by CaMKII (Zhang et al., 2019). Because the mutated lysines of ArcKR (K268/269) are positioned in close proximity to the Ser-260 pocket (Mabb et al., 2014; Wall et al., 2018; Zhang et al., 2019), we sought to determine whether Arc oligomerization potential could be altered when the proximal K268 and 269 ubiquitin sites were altered. Mutation of Lysine to Arginine is considered a conservative mutation that should have minimal impact on structure given that a positive charge is retained and its size is not dramatically changed. Thus, we used this conservative mutation and co-transfected HEK293 cells with N-terminal myc-tagged Arc (myc-ArcWT) or ArcKR (myc-ArcKR) and N-terminal GFP-tagged Arc (GFP-ArcWT) and GFP-ArcKR and performed a co-immunoprecipitation assay to compare the amount of Arc self-assembly. Immunoblotting following immunoprecipitation of myc-Arc and myc-ArcKR showed that ArcKR self-assembles more than ArcWT (Figures 4A, B). We next introduced a more dramatic mutation within Arc by mutating both of its ubiquitin sites at K268 and K269 to Alanine, a smaller amino acid that is more hydrophobic and uncharged (ArcKA) and can also not get ubiquitinated. Similar to ArcKR, we found that ArcKA was close to significant (*p* = 0.06) in having increased self-assembly compared to ArcWT (Supplementary Figure 1), providing evidence that Arc self-assembly is not dramatically affected by charge changes within this region.

The greater self-pulldown of Arc does not indicate oligomerization *per se*, as this method does not differentiate between direct and indirect interactions. Therefore, we next purified ArcWT and ArcKR from HEK 293 cells, which can support protein ubiquitination, and performed SDS-PAGE on the purified His-ArcWT and His-ArcKR proteins under reducing and non-reducing conditions (Figure 4C). Under non-reducing conditions, we observed a prominent Arc reactive band at ∼100 kDa in ArcWT, indicative of dimeric Arc, which represented a significantly higher fraction of total Arc protein compared to ArcKR (Figure 4D). Under reducing conditions, both ArcWT and ArcKR collapsed into their monomeric forms with prominent bands between 50 and 75 kDa (Figure 4C, white asterisk), with no significant difference in the proportion of total Arc signal contributed by these bands between mutants (Figure 4D). These findings cumulatively suggest that ArcKR has decreased dimer formation compared to ArcWT.

### 3.4. CaMKII and CamKII-dependent phosphorylation of Arc does not affect Arc ubiquitination

Protein phosphorylation has been demonstrated to be a pre-requisite for protein ubiquitination. Given the proximity of CamKII phosphorylation sites to Arc ubiquitination and the preference of Arc to assemble into high molecular weight complexes when it is not ubiquitinated, we speculated that CamKII could phosphorylate Arc to facilitate its ubiquitination. Here, we mutated the proximal CamKII Arc phosphorylation site to prevent its phosphorylation (S260A) and also generated a mutant to mimic CaMKII-dependent Arc phosphorylation (S260D) (Zhang et al., 2019). While overexpression of RNF216 led to robust Arc ubiquitination as previously described (Mabb et al., 2014), overexpresssion of CaMKIIa or CaMKIIb did not facilitate ubiquitination of WT Arc nor did mutating the S260 phosphorylation site have any effect on Arc ubiquitination status (Supplementary Figure 2). While it is possible that contamination with additional ubiquitinated proteins could be a limitation of this method, these findings suggest that CamKII does not promote Arc ubiquitination.

### 3.5. ArcKR neurons display regional alterations in Arc-CaMKII colocalization

**FIGURE 5:**
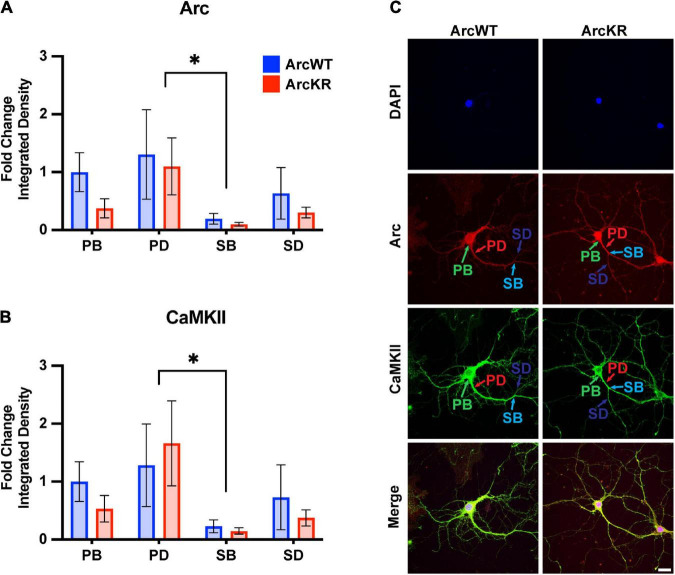
Arc and CaMKII are decreased at secondary branchpoints in ArcWT and ArcKR primary hippocampal neurons. **(A)** Fold change of Arc integrated densities for the indicated regions in ArcWT (blue, *N* = 7) and ArcKR (red, *N* = 5) primary hippocampal neurons relative to ArcWT PB. Primary branchpoint *n* = 12 WT, 11 ArcKR; primary dendrite *n* = 9 WT, 10 ArcKR; secondary branchpoint n = 10 WT, 11 ArcKR; secondary dendrites *n* = 10 WR, 11 ArcKR from 3 independently prepared neuron cultures. Two-way ANOVA, Genotype: *F*(1, 76) = 1.535, *p* = 0.2192, Region: *F*(3, 76) = 2.890, *p* = 0.0409, Interaction: *F*(3, 76) = 0.2099, *p* = 0.8892, Tukey’s post-hoc test, PD vs. SB: *p = 0.0270. **(B)** CaMKII integrated densities for indicated regions in panel **(A)**. Two-way ANOVA, Genotype: *F*(1, 76) = 0.3949, *p* = 0.7570, Region: *F*(3, 76) = 3.160, *p* = 0.0294, Interaction: *F*(3, 76) = 0.1961, *p* = 0.6592, Tukey’ *post-hoc* test PD vs. SB: *p = 0.0187. **(C)** Representative confocal images from ArcWT and ArcKR primary hippocampal neurons. Scale bar = 20 μm. Data represented as mean fold change relative to WT PB ± SEM.

**FIGURE 6:**
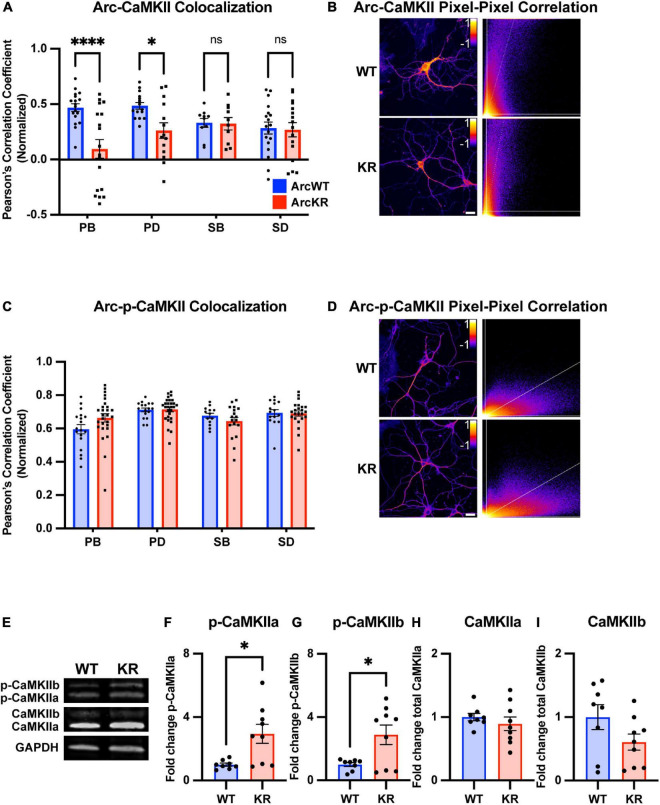
Global and regional alterations in Arc-CaMKII colocalization in ArcKR hippocampal neurons. **(A)** Pearson correlation coefficient between CaMKII and Arc in ArcWT (blue, *N* = 7) and ArcKR (red, *N* = 5) primary hippocampal neurons. Primary branchpoint *n* = 18 WT, 18 KR; primary dendrite *n* = 15 WT, 14 KR; secondary branchpoint *n* = 10 WT, 10 KR; secondary dendrites *n* = 18 WT, 16 KR from three independently prepared neuron cultures. Two-way ANOVA, Genotype: *F*(1, 111) = 12.54, *p* = 0.0006, Region: *F*(3, 111) = 1.166, *p* = 0.3261, Interaction: *F*(3, 111) = 4.475, *p* = 0.0053; Sidak’s *post-hoc* test WT vs. KR PB *p* < 0.0001, PD *p* = 0.0425, SB *p* > 0.9999, SD *p* = 0.9995. *p < 0.05, ^****^p < 0.0001, ns *p* > 0.05. **(B)** Representative pixel-pixel correlation heatmap between Arc and CaMKII for an ArcWT and ArcKR neuron (left) and corresponding pixel-pixel intensity plots with trendline (right). Scale bar = 20 μm. **(C)** Pearson correlation coefficient between p-CaMKII and Arc in ArcWT (blue, *N* = 7) and ArcKR (red, *N* = 9) primary hippocampal neurons. Primary branchpoint *n* = 18 WT, 27 KR; primary dendrite *n* = 17 WT, 28 KR; secondary branchpoint *n* = 13 WT, 18 KR; secondary dendrites *n* = 15 WT, 23 KR from 3 independently prepared neuron cultures. Two-way ANOVA Genotype: *F*(1, 151) = 0.3609, *p* = 0.5489, Region: *F*(3, 151) = 6.628, *p* = 0.0003, Interaction: *F*(3, 151) = 2.056, *p* = 0.1085; Sidak’s *post-hoc* test, PB vs. PD *p* = 0.0003, PB vs. SD p = 0.0181, **(D)** Representative pixel-pixel correlation heatmap between Arc and p-CaMKII for a WT and KR neuron (left) and corresponding pixel-pixel plots with trendline (right). Scale bar = 20 μm. **(E)** Representative Western blots for panels **(F–I)**. **(F)** Quantification of Western blot for p-CaMKIIα normalized to total CaMKIIα. Unpaired *t*-test, *t* = 3.015, df = 15, *p = 0.0087. **(G)** Quantification of Western blot for p-CaMKIIβ normalized to total CaMKIIβ. Unpaired *t*-test, *t* = 2.821, df = 15, *p = 0.0129. **(H)** Quantification of Western blot for total CaMKIIα. Unpaired *t*-test, *t* = 0.8324, df = 15, *p* = 0.4183. **(I)** Quantification of Western blot for total CaMKIIβ. Unpaired *t*-test, *t* = 1.712, df = 15, *p* = 0.1075. For panels **(A,C)**, data represented as mean fold change relative to WT PB ± SEM, for **(F–I)** data are represented as mean fold change relative to WT ± SEM.

Arc and CaMKII localization and colocalization within subregions of the neuron are critical for several signaling pathways that support learning and memory (Davies et al., 2007; Marsden et al., 2010), including localizing Arc to inactive synapses (Okuno et al., 2012). Given that our previous studies on Arc-CaMKII interactions were conducted in HEK293 cells, we sought to establish relationships of endogenously expressed Arc and CaMKII in primary hippocampal neurons. To discern potential changes in CaMKII localization and colocalization with Arc, we immunostained ArcWT and ArcKR primary hippocampal neuron cultures for CaMKII and Arc and imaged their distributions using confocal microscopy (Figures 5, 6).

Two-way ANOVA found no significant genotype difference in Arc protein levels (Figure 5A), however, there was a significant effect of region, with secondary branchpoints displaying lower levels than primary dendrites. Similarly, CaMKII levels were lower in the secondary branchpoints compared to primary dendrites (Figure 5B).

We next quantified the colocalization of Arc with total CaMKII from the same samples used in Figure 5. Pixel-pixel Pearson correlation values were lower in KR neurons compared to WT in PBs and PDs but not SBs or SDs (Figures 6A, B). Given the increase in ArcKR co-immunoprecipitation with both CamKIIa and CamKIIb constitutive active mutants, we stained separate ArcWT and ArcKR hippocampal neuron cultures for p-CaMKII and calculated the Arc-p-CaMKII colocalization. However, there were no significant genotype differences (Figures 6C, D).

Because of constraints in resolving isoform-specific changes in CaMKII levels with immunostaining, we used Western blotting to determine if CaMKIIa and CaMKIIb along with their respective levels of phosphorylation were altered in ArcKR hippocampal neuron cultures (Figure 6E). We observed that the proportion of the phosphorylated forms of CaMKIIa (p-CaMKIIa) and CaMKIIb (p-CaMKIIb) were greater in ArcKR neurons (Figures 6F, G) but there was no significant difference in the total expression of either isoform (Figures 6H, I). Taken together, these results suggest that while ArcKR protein and total CaMKII levels are unchanged in ArcKR neurons, ArcKR is biased toward interacting with p-CaMKII that may occur in a region-dependent manner.

### 3.6. The ER-bound protein calnexin co-immunoprecipitates more with ArcKR

**FIGURE 7:**
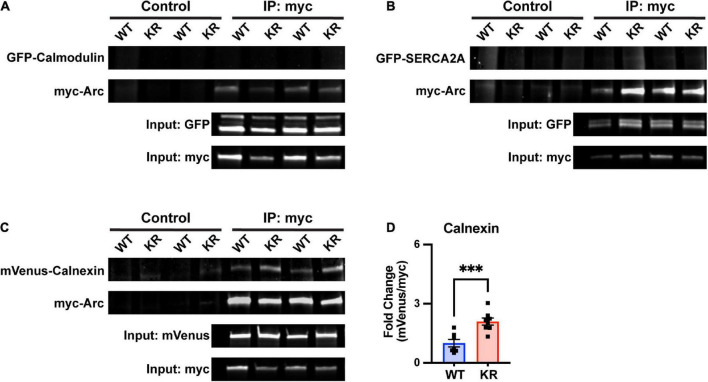
Enhanced co-immunoprecipitation of ArcKR with the ER membrane protein calnexin. **(A–C)** Representative blots after pulldown of myc-ArcWT or myc-ArcKR and immunoblotted for the indicated tagged proteins. **(D)** Increased co-immunoprecipitation of calnexin with ArcKR. WT *n* = 7, KR *n* = 8 from 2 separate transfection experiments. Unpaired *t*-test, *t* = 4.258, df = 13, ^***^p = 0.0009. Mean fold change relative to WT ± SEM.

As we are not aware of any literature implicating Arc levels or posttranslational modifications in regulating ER-mediated Ca^2+^ release, we performed co-immunoprecipitation assays with proteins known to be important for CaMKII or ER function. First, we considered the calcium-sensitive protein calmodulin which is required for CaMKII autophosphorylation (Lisman et al., 2002). However, we were unable to detect an interaction of GFP-tagged calmodulin with ArcWT or ArcKR (Figure 7A).

Arc has also been shown to directly interact with the cytoplasmic tail of the ER membrane-bound protein calnexin, although the functional consequence of this interaction is unknown (Myrum et al., 2017). Intriguingly, calnexin regulates the activity of the sarco/endoplasmic reticulum Ca^2+^-ATPase (SERCA), another ER-bound protein that sequesters Ca^2+^ in the lumen of the ER (Britzolaki et al., 2020). While SERCA2A did not co-immunoprecipitate with either ArcWT or ArcKR (Figure 7B), co-immunoprecipitation in HEK293 cells reaffirmed interaction of Arc with calnexin and, notably, revealed that ArcKR protein has an enhanced interaction with Calnexin compared to ArcWT (Figures 7C, D).

## 4. Discussion

Previous work has established that disrupting the temporal dynamics of Arc by reducing ubiquitination leads to increased AMPA receptor endocytosis, enhanced mGluR-LTD, and impairments of spatial reversal learning (Wall et al., 2018); however, the intracellular mechanisms by which these phenotypes occurred were unclear. Here we found that disrupting Arc ubiquitination leads to DHPG-induced alterations in ER Ca^2+^ release dynamics in all regions of the neuron except for secondary branchpoints. This phenotype was correlated with increased Arc-CaMKII interactions and Arc self-assembly. We also demonstrated that Arc ubiquitination and/or turnover may regulate its association with calnexin, providing a possible link between Arc ubiquitination levels and ER functionality.

As our previous work demonstrated that mGluR levels remained the same in ArcKR hippocampus relative to ArcWT (Wall et al., 2018), we hypothesized that dysregulation downstream of mGluR activation may be disrupted in ArcKR neurons. Indeed, we observed that ArcKR neurons release excess ER Ca^2+^ in response to DHPG. While Arc has previously been shown to interact with the cytoplasmic domain of the ER-associated protein calnexin via its central linker region (Myrum et al., 2017), to our knowledge there has never been a study directly implicating Arc in the modulation of ER Ca^2+^ release dynamics. Interestingly, our co-immunoprecipitation data suggest an increased association of ArcKR with calnexin. However, it is unclear if this increased interaction would lead to excess ER Ca^2+^ release in neurons. It is notable that the activity of SERCA, which is responsible for ER Ca^2+^ refilling (Berridge et al., 2003; Vandecaetsbeek et al., 2009), is modulated by and directly interacts with calnexin (Roderick et al., 2000; Lynes et al., 2013; Gutiérrez et al., 2020). Although we did not observe an interaction of ArcWT and ArcKR with SERCA, it is possible that a deficiency in Arc ubiquitination may indirectly reduce SERCA activity, perhaps by altering interactions between calnexin and SERCA. However, further work is necessary to test this hypothesis.

We observed that ArcKR had enhanced interactions with phosphomimetic forms of CaMKIIa and CaMKIIb. While baseline Ca^2+^ was not changed in ArcKR primary hippocampal neurons, phosphorylation of both CaMKIIa and CaMKIIb was elevated. Immunocytochemistry also demonstrated changes in Arc-CaMKII colocalization in select spatial regions of the neuron suggesting that alterations in Arc ubiquitination and self-assembly may have an effect on CaMKII interaction and activation profiles. CaMKII is important for mGluR-LTD magnitude (Mockett et al., 2011) and its interaction with Arc has previously been established (Okuno et al., 2012; Zhang et al., 2019). Mutations in the CaMKIIa T286 autophosphorylation site were found to block DHPG-induced LTD in hippocampal slices (Zhang et al., 2019). ArcKR neurons exhibit increased GluA2 surface trafficking (Wall et al., 2018), and activated CaMKII has been shown to work in complex with another calcium-regulated protein PICK1 (protein interacting with C-kinase 1) to facilitate GluA2 trafficking from the ER to the cell surface in an ER Ca^2+^ release-dependent manner (Lu et al., 2014). Notably, GluA2 undergoes Q/R editing, decreasing AMPA receptor Ca^2+^ permeability (Bowie and Mayer, 1995; Plant et al., 2006; Zhou et al., 2017), hinting at a potential compensatory effect of elevated calcium signaling in ArcKR neurons. Arc itself is known to interact with PICK1 via the PICK1 BAR domain, with the interaction and spatial distribution of the two proteins altered under depolarizing conditions (Goo et al., 2018); whether this interaction is influenced by the ArcKR mutation is not known.

We found that ArcKR has increased self-assembly compared to ArcWT. Using SDS-PAGE under non-reducing conditions, we found a decrease in dimer formation in ArcKR compared to ArcWT. Interestingly, Arc multermerization is negatively regulated by CaMKII-dependent phosphorylation at Ser 260. Mutation of CaMKII phosphorylation sites on Arc leads to enhanced mGluR-LTD and causes impairments in the acquisition of fear induced memories (Zhang et al., 2019). Mirroring that work, we also found that ArcKR mice had enhanced mGluR-LTD (Wall et al., 2018) and impaired fear memories (data not shown). Given these correlates, we hypothesized that CaMKII-dependent Arc phosphorylation at S260 could serve as a prerequisite for facilitating Arc ubiquitination, which would decrease its ability to oligomerize and temper ER-mediated Ca^2+^ release. However, overexpression of CaMKII in HEK293 cells and mutating the CaMKII-dependent phosphorylation sites on Arc had no effect on Arc ubiquitination. These findings suggest two possibilities. First, Arc ubiquitination may be a pre-requisite for CaMKII-dependent phosphorylation. The ubiquitination of Arc at K268 and K269 could allow for accessibility of the S260 site to CaMKII, which cumulatively would reduce its oligomerization to control mGluR-LTD magnitude (Zhang et al., 2019). On the other hand, Arc ubiquitination could sever its interaction with CaMKII, leading to its degradation or allow for it to function independent of CaMKII complexes. This could explain why the ubiquitin Arc defective mutant (ArcKR) has increased interactions with CaMKIIb and constitutive active forms of CaMKII. Regardless, these proposed mechanisms could be a way in which neurons provide regional specificity along dendritic regions to fine-tune synaptic strength in response to select synaptic inputs.

Arc-dependent intracellular Ca^2+^ dysregulation carries important implications for behavioral output. ArcKR mice exhibit impaired spatial reversal learning on a Barnes maze task which was associated with enhanced mGluR-LTD (Wall et al., 2018). Further work is aimed at determining whether this behavioral deficit is also due in part to other ArcKR effects observed in this study. For example, dysregulation of CaMKII phosphorylation has been shown to affect strategy selection on the Barnes maze (Bach et al., 1995). Furthermore, N-methyl-D-aspartate receptor-mediated LTD is required for behavioral flexibility on a similarly spatial learning-dependent Morris water maze task (Nicholls et al., 2008; Kim et al., 2011). Prevention of LTD induction and reduction of AMPA receptor endocytosis caused by stabilization of the adhesion molecule β-catenin causes similar deficits in reversal learning on the water maze (Mills et al., 2014). Enhanced Arc translation is found to occur in Fragile X-syndrome model mice, which also display enhanced mGluR-LTD and increased AMPA receptor endocytosis (Hou et al., 2006; Nakamoto et al., 2007; Cheng et al., 2017). It is possible that this model has increased DHPG-induced ER-Ca^2+^ release similar to our ArcKR mice. Tempering ER-Ca^2+^ release via increases in Arc ubiquitination might serve as an alternative mechanism that could restore excessive mGluR-LTD in this mouse model.

Cumulatively, our ArcKR model suggests that the cooperation of mGluR-LTD within an optimal magnitude, threshold, and temporal range are necessary for intracellular signaling events that control behavioral outputs – whether some of these are regulated by Arc ubiquitination, Arc-CaMKII interaction, and regulation of ER Ca^2+^ release is a subject for future research.

## Data availability statement

The raw data supporting the conclusions of this article will be made available by the authors, without undue reservation.

## Ethics statement

This animal study was reviewed and approved by Georgia State University Institutional Animal Care and Use Committee.

## Author contributions

MG designed the study, performed the experiments, and wrote the manuscript. DY, WW, ZA, CM, and BD performed the experiments. NF and JY contributed to the study design. AM designed the study, edited the manuscript, and contributed to the manuscript writing. All authors contributed to the article and approved the submitted version.

## Supplementary material

Supplementary Figure 1 A trend in ArcKA self-assembly relative to ArcWT. **(A)** Representative Western blots after pulldown of myc-ArcWT or myc-ArcKA with an anti-myc antibody and immunoblotted with anti-GFP to detect GFP-ArcWT or GFP-ArcKA. **(B)** Quantification of GFP-ArcWT or GFP-ArcKA after pulldown with myc-Arc or myc-ArcKA. Unpaired *t*-test with Welch’s correction for unequal variances, *t* = 2.583 df = 3.138, *p* = 0.06. Mean fold change relative to WT ± SEM.

Supplementary Figure 2 CaMKII and CaMKII-dependent Arc phosphorylation does not alter Arc ubiquitination status. RIPA cell lysates from HEK293 cells transfected with myc-Arc, myc-Arc S260A or myc-Arc S260D with GFP-CaMKIIa (IIa) or GFP-CaMKIIb (IIb). Samples were immunoprecipitated (IP) with an anti-myc antibody followed by immunoblot (IB) analysis using an anti-ubiquitin (Ub) antibody to detect ubiquitinated Arc. The E3 ubiquitin ligase, GFP-RNF216 was used as a positive control for myc-Arc ubiquitination.
