# Supplementary figures and images for: Arc ubiquitination regulates endoplasmic reticulum-mediated Ca2+ release and CaMKII signaling
Source: Front Cell Neurosci. 2023 Mar 14;17:1091324. doi: 10.3389/fncel.2023.1091324 (PMC10043188; doi:10.3389/fncel.2023.1091324)

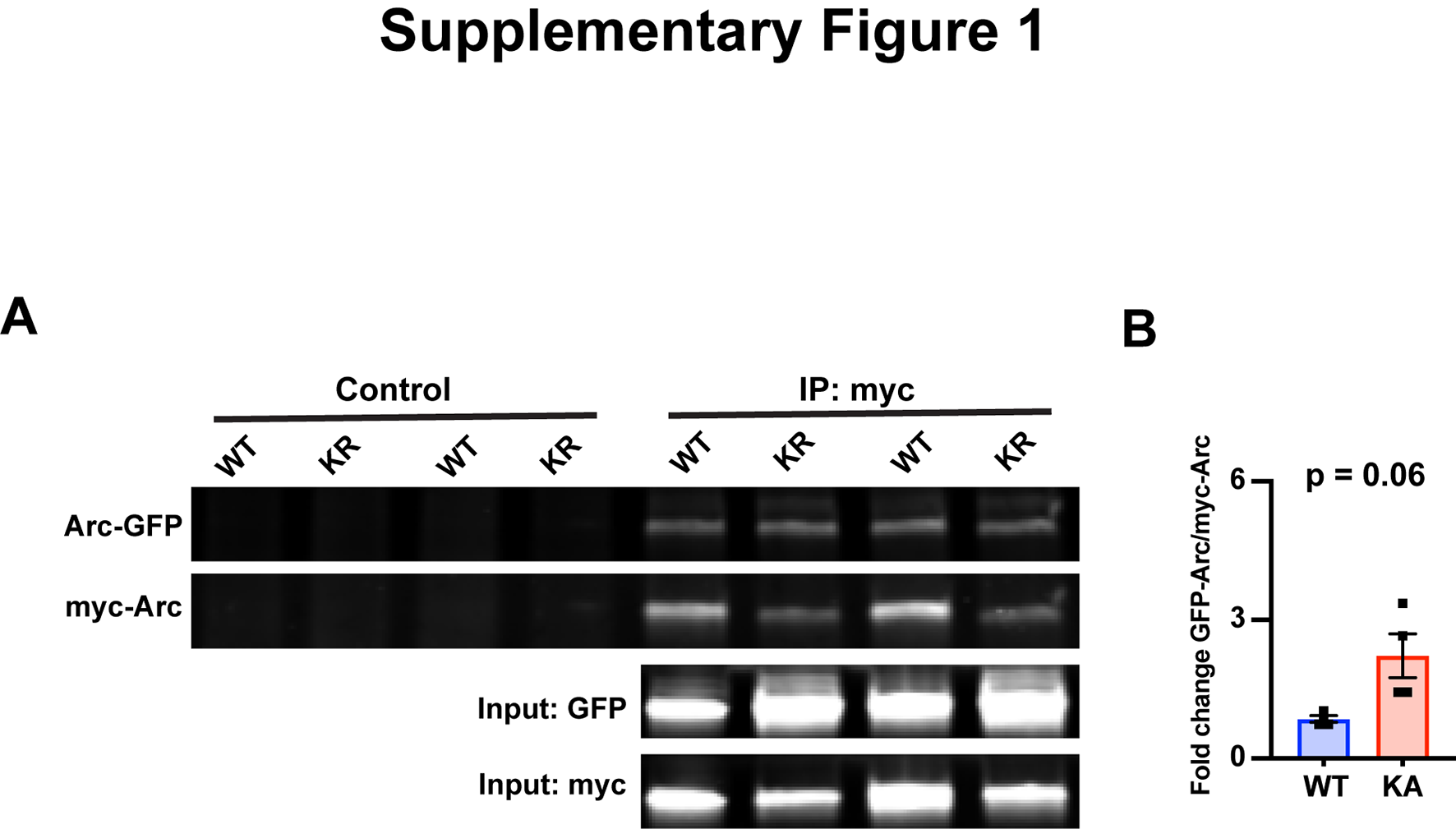

Supplement: Supplementary Figure 1 — A trend in ArcKA self-assembly relative to ArcWT. (A) Representative Western blots after pulldown of myc-ArcWT or myc-ArcKA with an anti-myc antibody and immunoblotted with anti-GFP to detect GFP-ArcWT or GFP-ArcKA. (B) Quantification of GFP-ArcWT or GFP-ArcKA after pulldown with myc-Arc or myc-ArcKA. Unpaired t-test with Welch’s correction for unequal variances, t = 2.583 df = 3.138, p = 0.06. Mean fold change relative to WT ± SEM. [file Image_1.TIF]

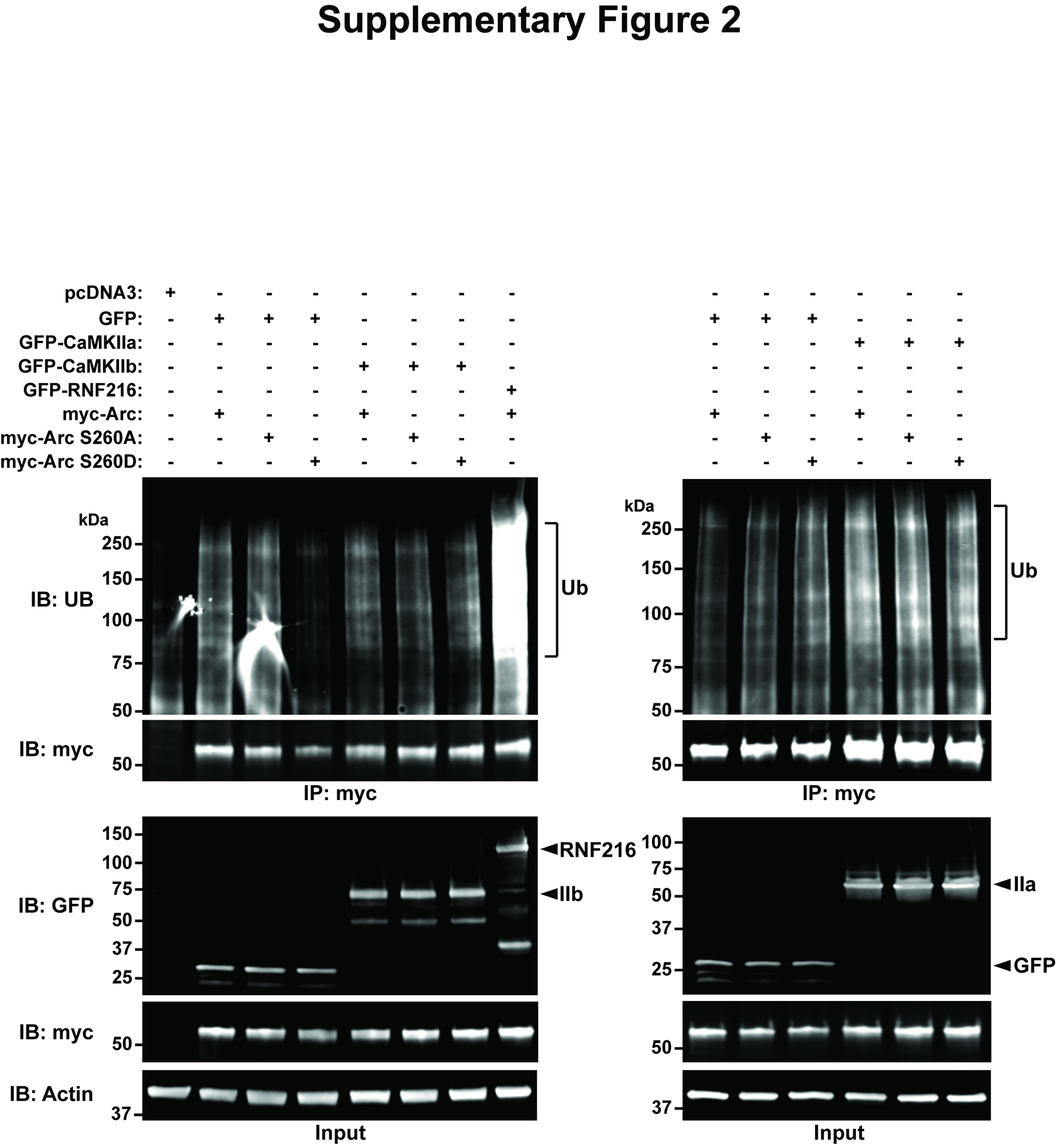

Supplement: Supplementary Figure 2 — CaMKII and CaMKII-dependent Arc phosphorylation does not alter Arc ubiquitination status. RIPA cell lysates from HEK293 cells transfected with myc-Arc, myc-Arc S260A or myc-Arc S260D with GFP-CaMKIIa (IIa) or GFP-CaMKIIb (IIb). Samples were immunoprecipitated (IP) with an anti-myc antibody followed by immunoblot (IB) analysis using an anti-ubiquitin (Ub) antibody to detect ubiquitinated Arc. The E3 ubiquitin ligase, GFP-RNF216 was used as a positive control for myc-Arc ubiquitination. [file Image_2.TIF]
